# A protective role of ABCA5 in response to elevated sphingomyelin levels in Parkinson’s disease

**DOI:** 10.1038/s41531-024-00632-2

**Published:** 2024-01-11

**Authors:** YuHong Fu, Russell Pickford, Jasmin Galper, Katherine Phan, Ping Wu, Hongyun Li, Young-Bum Kim, Nicolas Dzamko, Glenda M. Halliday, Woojin Scott Kim

**Affiliations:** 1 Brain and Mind Centre, The University of Sydney, Sydney, NSW Australia; 2 School of Medical Sciences, The University of Sydney, Sydney, NSW Australia; 3 Bioanalytical Mass Spectrometry Facility, University of New South Wales, Sydney, NSW Australia; 4 Division of Endocrinology, Diabetes, and Metabolism, Beth Israel Deaconess Medical Center and Harvard Medical School, Boston, MA USA

**Keywords:** Parkinson's disease, Metabolomics

## Abstract

Parkinson’s disease (PD) is a chronic neurodegenerative disorder that affects the motor system. Increasing evidence indicates that lysosomal dysfunction is pivotal in the pathogenesis of PD, typically characterized by dysregulation of sphingolipids in lysosomes. ATP-binding cassette subfamily A member 5 (ABCA5) is a lysosomal transporter that mediates the removal of excess sphingomyelin from lysosomes. We therefore investigated whether the expression levels of ABCA5 are associated with sphingomyelin levels and α-synuclein pathology in PD. Firstly, we undertook a comprehensive assessment of the six sphingolipid classes that are part of the lysosomal salvage pathway in the disease-affected amygdala and disease-unaffected visual cortex using liquid chromatography-mass spectrometry. We found that sphingomyelin levels were significantly increased in PD compared to controls and correlated with disease duration only in the amygdala, whereas, the five other sphingolipid classes were slightly altered or unaltered. Concomitantly, the expression of ABCA5 was upregulated in the PD amygdala compared to controls and correlated strongly with sphingomyelin levels. Using neuronal cells, we further verified that the expression of ABCA5 was dependent on cellular levels of sphingomyelin. Interestingly, sphingomyelin levels were strongly associated with α-synuclein in the amygdala and were related to α-synuclein expression. Finally, we revealed that sphingomyelin levels were also increased in PD plasma compared to controls, and that five identical sphingomyelin species were increased in both the brain and the plasma. When put together, these results suggest that in regions accumulating α-synuclein in PD, ABCA5 is upregulated to reduce lysosomal sphingomyelin levels potentially as a protective measure. This process may provide new targets for therapeutic intervention and biomarker development for PD.

## Introduction

Parkinson’s disease (PD) is the most prevalent neurodegenerative movement disorder. Its hallmark pathology is neuronal inclusions composed of accumulated α-synuclein associated with lipids^1,2^. Sphingolipid is a class of lipids that contain the sphingoid backbone that include ceramides, glucosylceramides (*aka* glucocerebroside), sphingomyelins and glucosylsphingosines. Sphingolipid metabolism or transport is increasingly recognized to be important in PD pathogenesis, with the glucocerebroside hydrolysis gene *GBA1* being the strongest genetic risk factor for PD^3^. Glucocerebrosidase activity is reduced in both sporadic PD^4^ and PD with *GBA1* mutations^5^, and glucosylsphingosine is strongly associated with α-synuclein pathology in specific regions of PD brain^5^. Interestingly, homozygote forms of *GBA1* mutations cause Gaucher’s disease, a lysosomal lipid storage disease characterized by the accumulation of glucosylceramide^6^.

The transport of lipids, including sphingolipids is mediated by a group of proteins called ATP-binding cassette subfamily A (ABCA) transporters. These membrane-bound proteins are responsible for the efflux (i.e. removal) of excess lipids out of cells or organelles, and in recent years, they have been identified as pivotal in the regulation of many neurodegenerative processes^7,8^. For example, the prototype ABCA1 mediates the efflux of cholesterol out of cells that regulate the processing of amyloid precursor protein to amyloid-β peptides^9–13^. Deletion of ABCA1 in amyloidogenic mice significantly increases the formation of amyloid-β plaques^14^, indicating that ABCA1 is neuroprotective against cellular processes associated with Alzheimer’s disease.

**Fig. 1:**
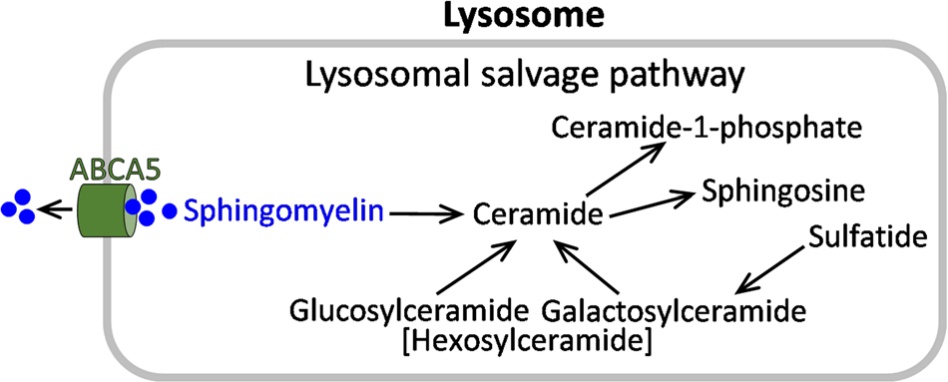
Sphingomyelin and other sphingolipids are part of the lysosomal salvage pathway. ATP-binding cassette subfamily A member 5 (ABCA5) mediates the efflux of excess sphingomyelin from lysosomes.

Despite the apparent importance of ABCA transporters in neurodegenerative diseases, very little is known about the role of ABCA transporters in the pathological processes of PD. In 2009, a genome-wide association study of PD showed that ABCA5 SNPs are associated (*P* = 2.53 × 10^−5^) with a reduced risk for PD^15^. The exact function of ABCA5 is unclear, although it is thought to mediate the efflux of sphingomyelin^16^. A clue to the possible function of ABCA5 in PD comes from a 2005 study, which showed that deletion of *Abca5* in mice replicated a lysosomal storage disorder similar to Gaucher’s disease^17^. Abca5 is highly expressed in the mouse brain and is localized to the lysosomes^17^. Sphingomyelin, along with other sphingolipids, is a part of the lysosomal salvage pathway (LSP, Fig. 1), which is altered in PD^18^. The role of ABCA5 in PD pathogenesis is unknown and no pathogenic mechanism has yet been proposed for it reducing the risk of PD, providing a key rationale for this study.

In this study, we undertook a comprehensive assessment of sphingomyelin, as well as other sphingolipids that are a part of the LSP, in PD brain. We investigated the functional role of ABCA5, how it is transcriptionally regulated, and how sphingomyelin could be pathologically linked to α-synuclein. We also assessed sphingomyelin in PD plasma and identified sphingomyelin species that could potentially serve as peripheral biomarkers for PD.

## Results

### Assessment of α-synuclein pathology and neuronal loss in PD amygdala and visual cortex

**Fig. 2:**
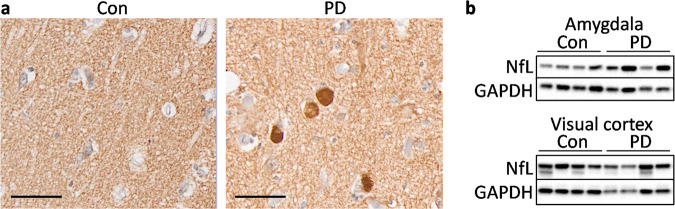
Assessment of neuropathology and neurodegeneration in PD tissues. **a** α-Synuclein deposits are present in PD amygdala and absent in control amygdala. Scale bar = 50 µm. **b** The neurofilament light (NfL) protein is unaltered in PD amygdala and visual cortex compared to controls as measured by Western blotting.

Assessment of Lewy pathology (using α-synuclein immunohistochemistry) and neurodegeneration (using neurofilament light (NfL) Western blotting) revealed numerous α-synuclein deposits in the PD amygdala but not controls (Fig. 2a) or the visual cortex. This is consistent with the distribution of Lewy pathology in PD^19^. NfL levels were unaltered in the PD tissues (Fig. 2b), suggesting neuronal integrity within these tissue structures in these samples. This is consistent with limited cell loss in either the amygdala or visual cortex in PD brains in those without other comorbid pathologies^19^.

### Sphingomyelin levels are increased in PD amygdala

**Fig. 3:**
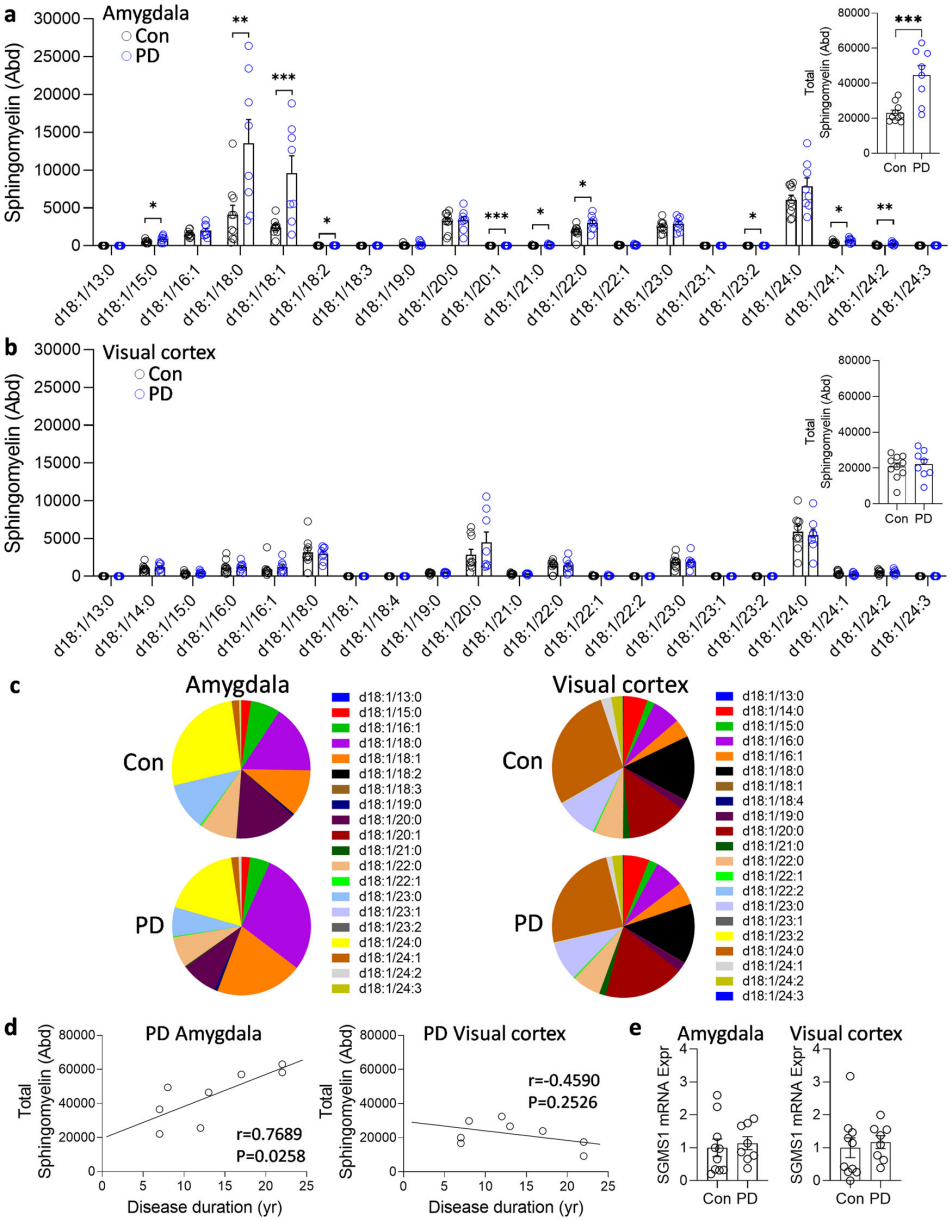
Liquid chromatography-mass spectrometry analysis of sphingomyelin species in sporadic PD brain tissues. **a** Ten sphingomyelin species were significantly increased in the PD amygdala compared to controls; (inset) the total sphingomyelin levels obtained from the addition of all sphingomyelin species were also significantly increased in the PD amygdala. **b** None of the sphingomyelin species were significantly altered in the PD visual cortex compared to controls; (inset) the total sphingomyelin levels were not significantly altered in the PD visual cortex. **c** The distribution of sphingomyelin species in each lipidome. **d** The total sphingomyelin levels correlated with disease duration in the amygdala only. **e** The expression of sphingomyelin synthase (SGMS1) was not altered in either of the two regions. Data represent mean and S.E.M. as error bars. *P < 0.05, **P < 0.01, ***P < 0.005.

As detailed in the introduction, sphingomyelin is a part of the LSP (Fig. 1) which is dysregulated in PD. Here, we undertook a comprehensive analysis of sphingomyelin in the disease-affected amygdala and disease-unaffected visual cortex of sporadic PD and control brain using liquid chromatography-mass spectrometry (LC-MS) and LipidSearch software. Ten of the twenty detected sphingomyelin species were significantly increased in the PD amygdala compared to controls (Fig. 3a), but not the visual cortex (Fig. 3b). The total sphingomyelin levels, obtained from the addition of all sphingomyelin species, were also significantly increased in the PD amygdala (Fig. 3a inset) but not the visual cortex (Fig. 3b inset). We also analyzed the distribution of sphingomyelin species in each lipidome and found that five species (d18:1/16:1, d18:1/20:0, d18:1/20:1, d18:1/23:0, d18:1/24:0) were significantly altered in PD amygdala only (Fig. 3c). Total sphingomyelin levels also correlated with disease duration in the amygdala only (Fig. 3d). The expression of sphingomyelin synthase (*SGMS1*), the gene responsible for sphingomyelin synthesis, was also assessed and it was not altered in either of the two regions (Fig. 3e), indicating that the increase in sphingomyelin in PD was due to sphingomyelin processing/turnover and not synthesis.

**Fig. 4:**
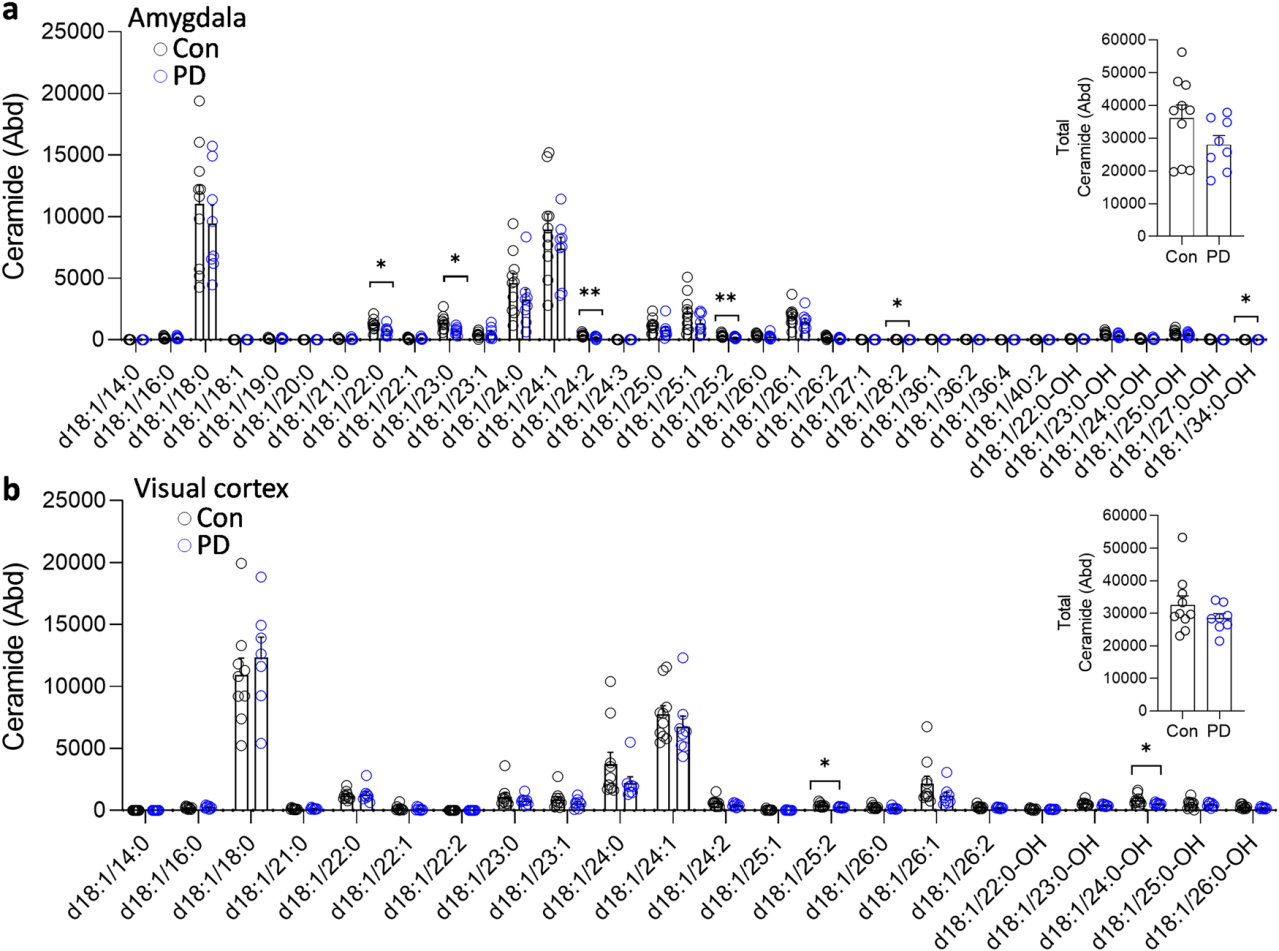
Liquid chromatography-mass spectrometry analysis of ceramide species in PD brain tissues. **a** Six ceramide species were decreased in the PD amygdala compared to controls; (inset) the total ceramide levels were not significantly altered in the PD amygdala. **b** Two ceramide species were decreased in the PD visual cortex compared to controls; (inset) the total ceramide levels were not significantly altered in the PD visual cortex. Data represent mean and S.E.M. as error bars. *P < 0.05, **P < 0.01.

**Fig. 5:**
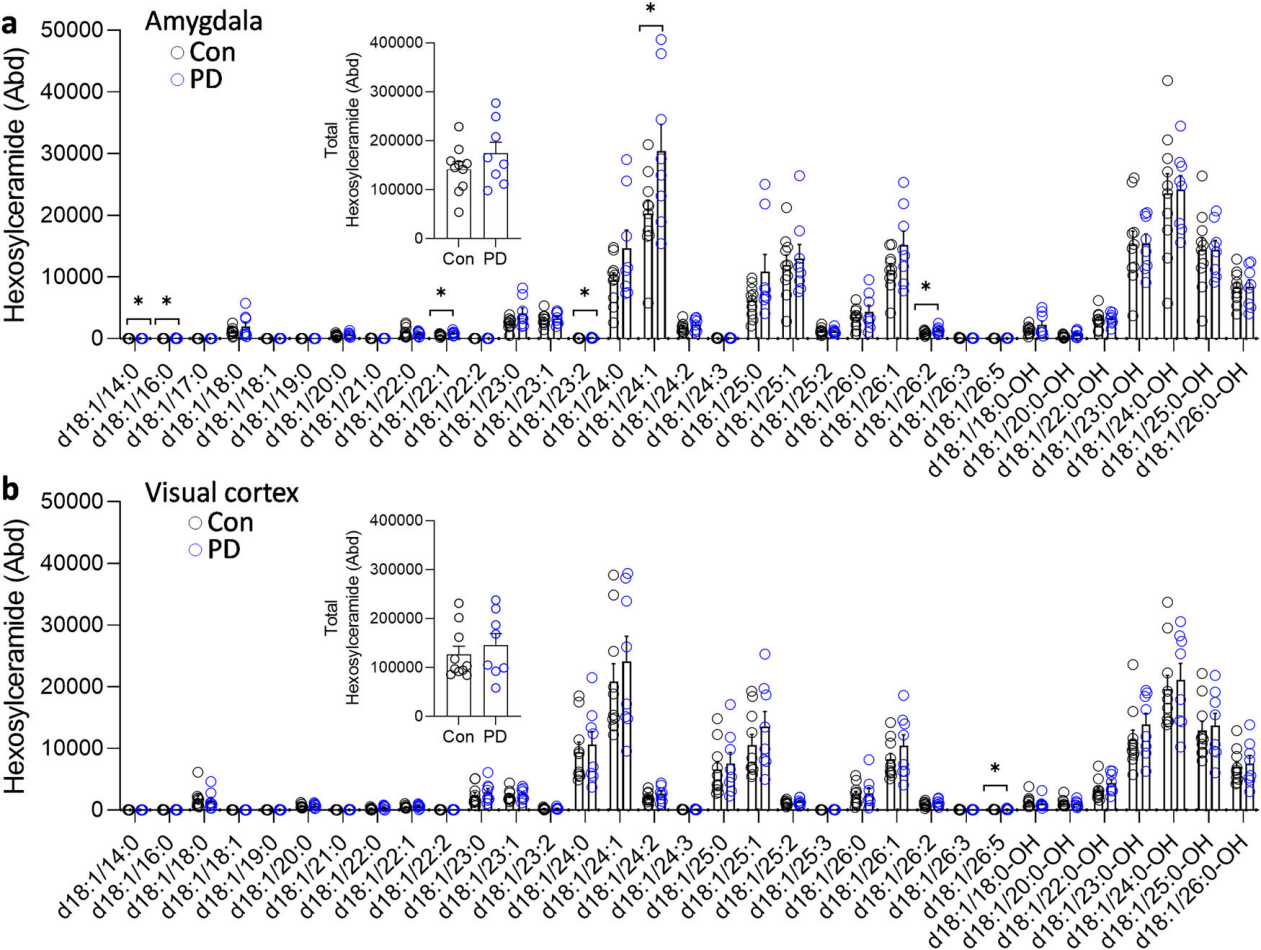
Liquid chromatography-mass spectrometry analysis of hexosylceramide species in PD brain tissues. **a** Six hexosylceramide species were increased in the PD amygdala compared to controls; (inset) the total hexosylceramide levels were not significantly altered in the PD amygdala. **b** One ceramide species was increased in the PD visual cortex compared to controls; (inset) the total ceramide levels were not significantly altered in the PD visual cortex. Data represent mean and S.E.M. as error bars. *P < 0.05.

**Fig. 6:**
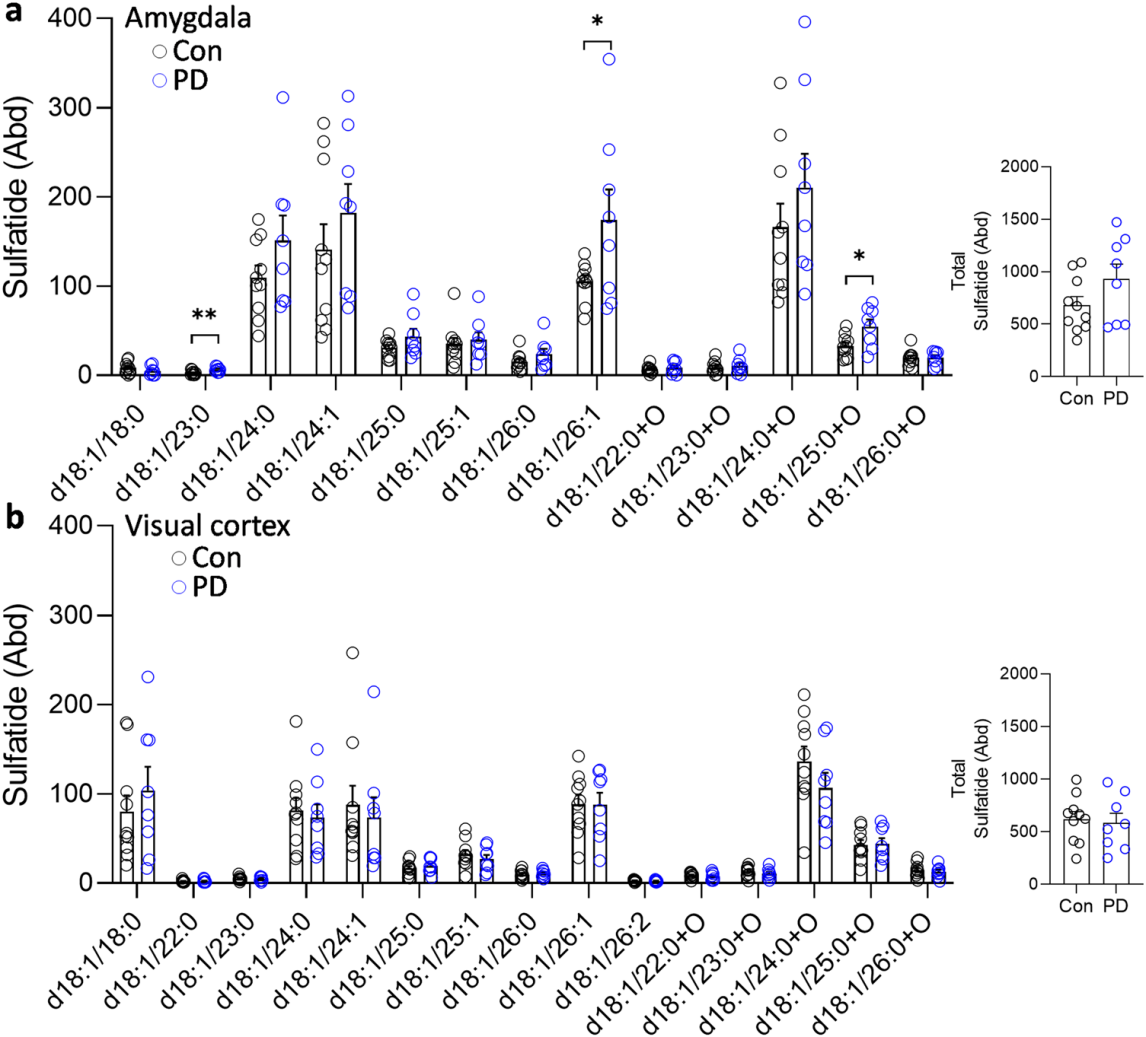
Liquid chromatography-mass spectrometry analysis of sulfatide species in PD brain tissues. **a** Three sulfatide species were increased in the PD amygdala compared to controls; (inset) the total sulfatide levels were not significantly altered in the PD amygdala. **b** None of the sulfatide species were altered in the PD visual cortex compared to controls; (inset) the total sulfatide levels were not significantly altered in the PD visual cortex. Data represent mean and S.E.M. as error bars. *P < 0.05, **P < 0.01.

**Fig. 7:**
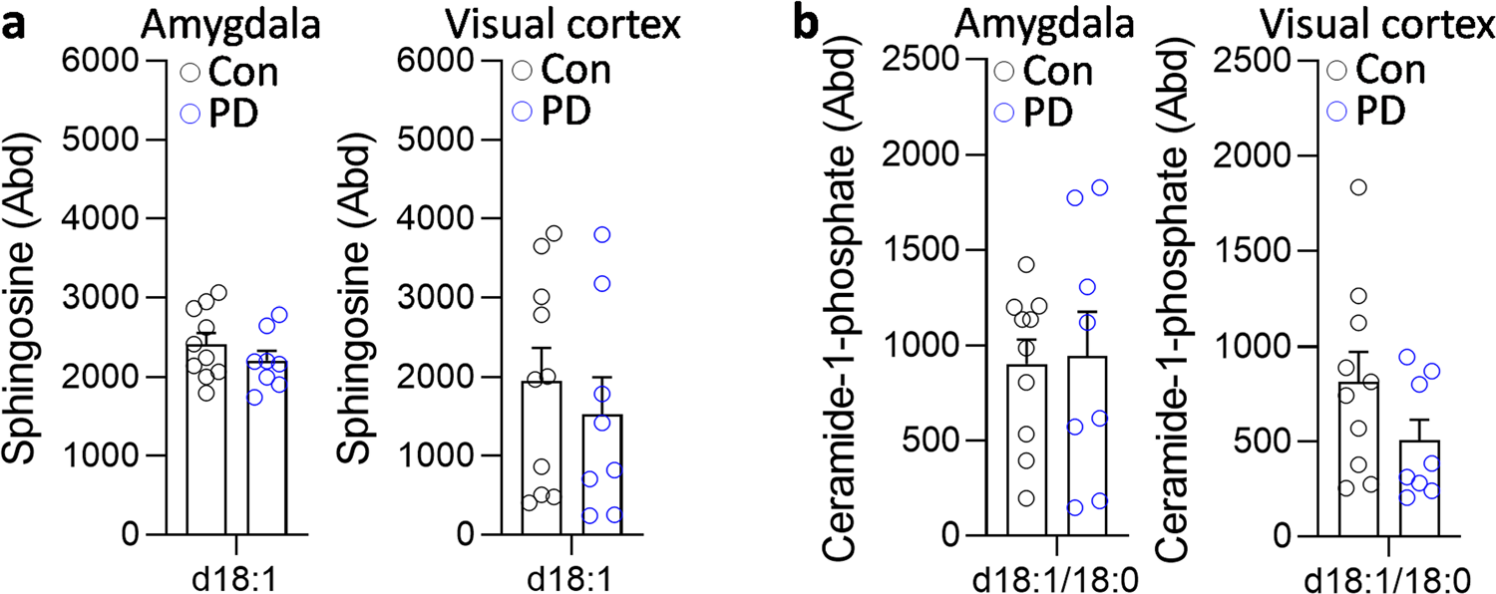
Liquid chromatography-mass spectrometry analysis of sphingosine and ceramide-1-phosphate in PD brain tissues. **a** Sphingosine and **b** ceramide-1-phosphate were not significantly altered in the PD amygdala and visual cortex compared to controls. Data represent mean and S.E.M. as error bars.

To determine whether the changes were restricted to sphingomyelin, the five other sphingolipids that are a part of the LSP (Fig. 1) were analyzed. Six ceramide species (Fig. 4) were decreased, and six hexosylceramide species (Fig. 5) and three sulfatide species (Fig. 6) were increased in the PD amygdala compared to controls. There were no significant changes in sphingosine nor ceramide-1-phosphate in the PD amygdala compared to controls (Fig. 7). In contrast, little or no changes occurred in the PD visual cortex (Figs. 4–7). Importantly, the total levels of each of the five sphingolipids were not altered in either of the two regions (Figs. 4–7). When put together, these results suggest that sphingomyelin is altered the most in PD amygdala.

### Upregulation of ABCA5 in response to increased sphingomyelin levels in PD

**Fig. 8:**
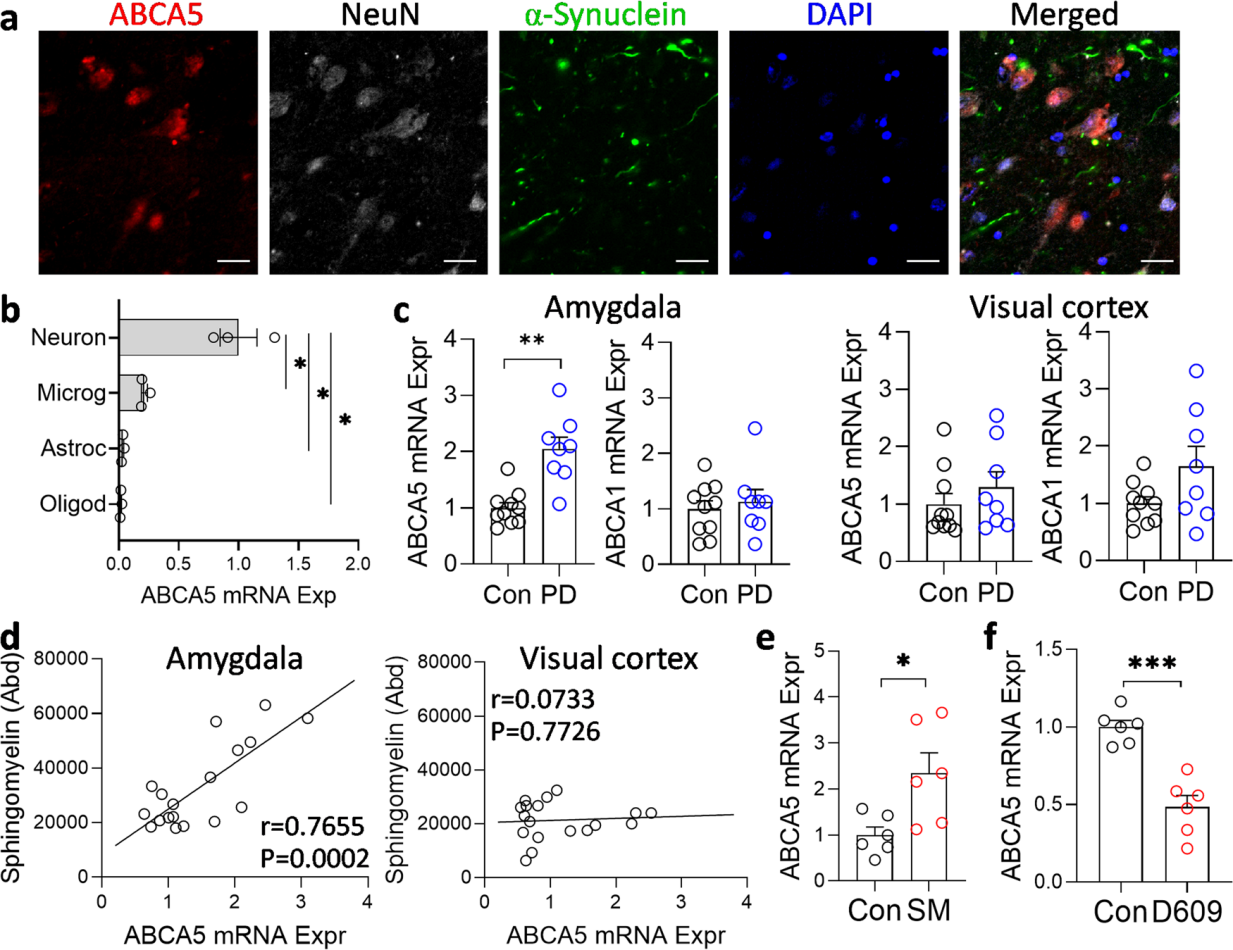
Upregulation of ABCA5 expression in PD amygdala. **a** ABCA5 was widely expressed in the neurons of PD amygdala as assessed by immunofluorescence. Scale bar = 20 µm. **b** ABCA5 was highly expressed in the primary human neurons. **c** ABCA5 was upregulated in the PD amygdala compared to controls and unchanged in the PD visual cortex; ABCA1 (negative control) was unaltered in both tissues. **d** ABCA5 expression correlated strongly with sphingomyelin levels in the amygdala only. **e** ABCA5 expression was increased when SK-N-SH neuronal cells were treated with exogenous sphingomyelin. **f** Conversely, ABCA5 expression was decreased when the cells were treated with the SGMS1 inhibitor D609. Data represent mean and S.E.M. as error bars. *P < 0.05, **P < 0.005, ***P < 0.0005.

While ABCA5 is expressed in lysosomes^17^, its location in the brain has not been assessed. We therefore analyzed ABCA5 in the amygdala by immunofluorescence and in primary brain cells by qPCR. In brain tissue, ABCA5 protein was present throughout the neuronal cytoplasm (Fig. 8a), and across different brain cells in culture, neurons had the highest expression (Fig. 8b). Since sphingomyelin levels were increased in PD and ABCA5 removes excess sphingomyelin from lysosomes (Fig. 1), we were interested in whether the expression of ABCA5 is altered in PD brain. We assessed the expression of ABCA5 in the same tissue samples as the lipid measurement. We also assessed the expression of ABCA1, which is the ABCA subfamily prototype that does not transport sphingomyelin, as a negative control. We found that ABCA5 expression was significantly increased in the PD amygdala compared to controls (Fig. 8c) and correlated strongly with sphingomyelin in the amygdala only (Fig. 8d), whereas ABCA1 was unaltered (Fig. 8c).

To further investigate the relationship between sphingomyelin and ABCA5, we then carried out a series of in vitro studies using SK-N-SH neuronal cells. Firstly, we treated the cells with exogenous sphingomyelin (i.e. increases in cellular sphingomyelin) and found that the expression of ABCA5 was increased (Fig. 8e). Conversely, when the cells were treated with the SGMS1 inhibitor D609 (i.e. decreases in cellular sphingomyelin) the expression of ABCA5 was decreased (Fig. 8f). When put together, these results suggest that the expression of ABCA5 is regulated in response to levels of sphingomyelin.

### α-Synuclein expression is associated with sphingomyelin levels

**Fig. 9:**
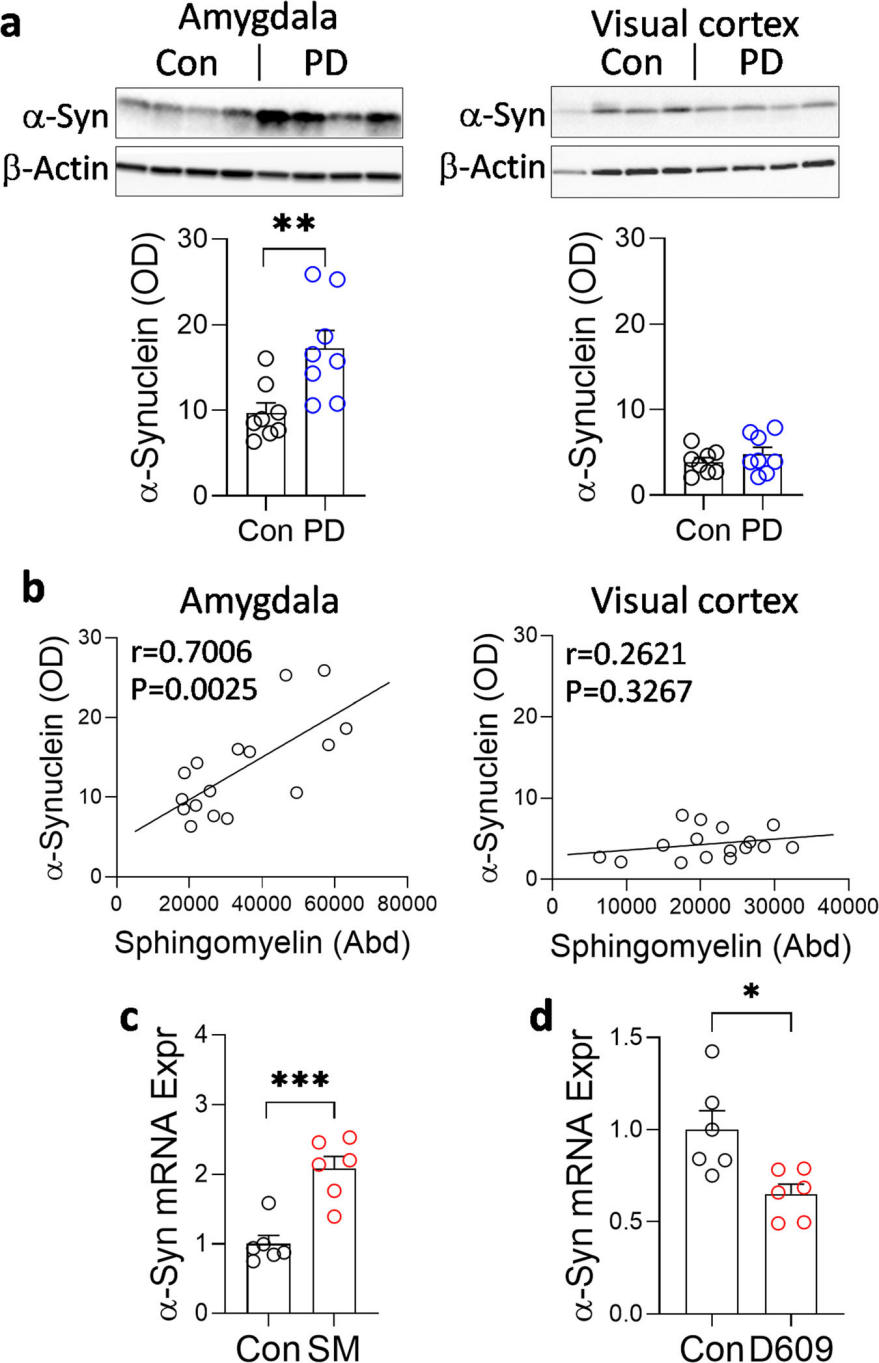
α-Synuclein expression is associated with sphingomyelin levels. **a** α-Synuclein protein load was elevated in the PD amygdala compared to controls and unchanged in the visual cortex. **b** α-Synuclein protein load correlated strongly with sphingomyelin levels in the amygdala only. **c** α-Synuclein expression was increased when SK-N-SH neuronal cells were treated with exogenous sphingomyelin. **d** Conversely, α-synuclein expression was decreased when the cells were treated with the SGMS1 inhibitor D609. Data represent mean and S.E.M. as error bars. *P < 0.05, ***P < 0.0005.

To understand the possible contribution of sphingomyelin to PD pathology, we examined its link, if any, to α-synuclein. Firstly, we assessed the amount of α-synuclein protein in the same tissue samples as the lipid measurement and found that it was increased in the PD amygdala compared to controls and unchanged in the visual cortex (Fig. 9a). Importantly, there was a strong correlation between α-synuclein protein and sphingomyelin levels in the amygdala only (Fig. 9b). When SK-N-SH neuronal cells were treated with exogenous sphingomyelin, α-synuclein expression was increased (Fig. 9c). Conversely, when the cells were treated with the SGMS1 inhibitor D609, α-synuclein expression was decreased (Fig. 9d). When put together, these results suggest that α-synuclein expression levels are associated with sphingomyelin levels and that increased α-synuclein expression results in increased protein levels which also relate to increased sphingomyelin levels.

### ABCA5 transcription is regulated by PPARγ

**Fig. 10:**
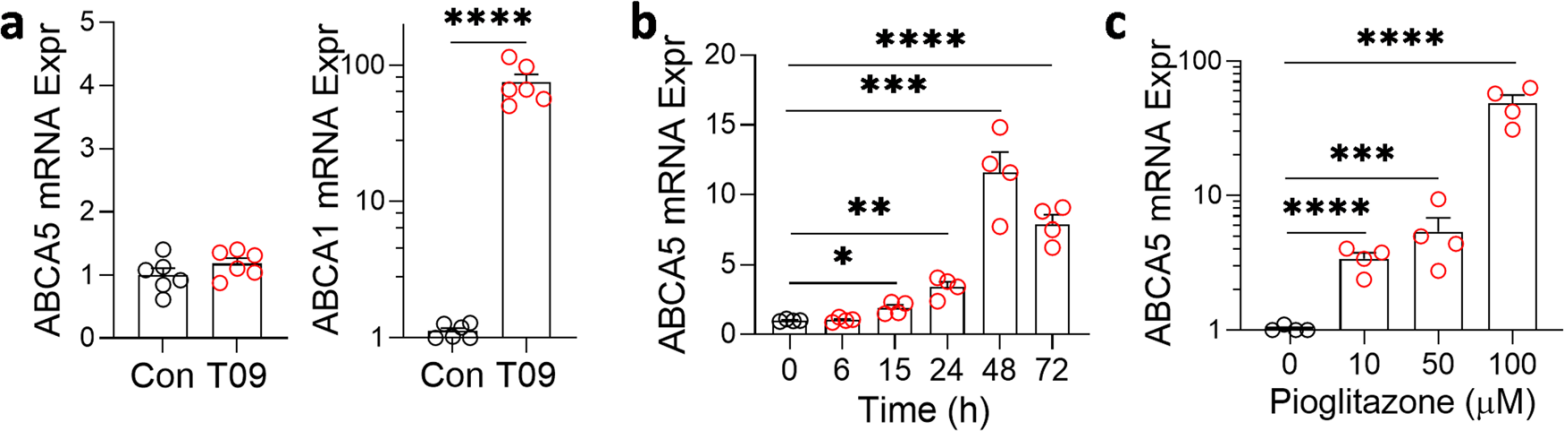
Transcriptional regulation ABCA5. **a** ABCA5 transcription was unaltered when SK-N-SH neuronal cells were treated with the LXR ligand T0901317 + RXR ligand 9-cis retinoic acid (T09); ABCA1 (positive control) transcription was upregulated. **b** ABCA5 transcription was upregulated when SK-N-SH neuronal cells were treated with the PPARγ ligand pioglitazone (10 µM) + RXR ligand 9-cis retinoic acid in a time-dependent manner. **c** ABCA5 transcription was upregulated when the cells were treated with increasing concentrations of the PPARγ ligand pioglitazone + RXR ligand 9-cis retinoic acid. Data represent mean and S.E.M. as error bars. *P < 0.05, **P < 0.005, ***P < 0.0005, ****P < 0.00005.

Next, we investigated how ABCA5 is transcriptionally regulated as this could provide an avenue for controlling cellular sphingomyelin levels. Clues come from the fact that a few members of the ABCA subfamily are transcriptionally regulated by the nuclear receptor family of transcription factors, i.e. liver X receptor (LXR), retinoid X receptor (RXR) and peroxisome proliferator-activated receptor-γ (PPARγ). To test whether ABCA5 is similarly regulated, we treated SK-N-SH neuronal cells with the LXR ligand T0901317, RXR ligand 9-cis retinoic acid and PPARγ ligand pioglitazone, and assessed ABCA5 transcription by qPCR. As a positive control, ABCA1 transcription was also assessed. The application of the LXR ligand T0901317 + RXR ligand 9-cis retinoic acid had no effect on ABCA5 transcription, only on ABCA1 (Fig. 10a). In contrast, the application of the PPARγ ligand pioglitazone + RXR ligand 9-cis retinoic acid promoted ABCA5 transcription in a time- (Fig. 10b) and dose-dependent manner (Fig. 10c).

### Sphingomyelin levels are increased in PD plasma

**Fig. 11:**
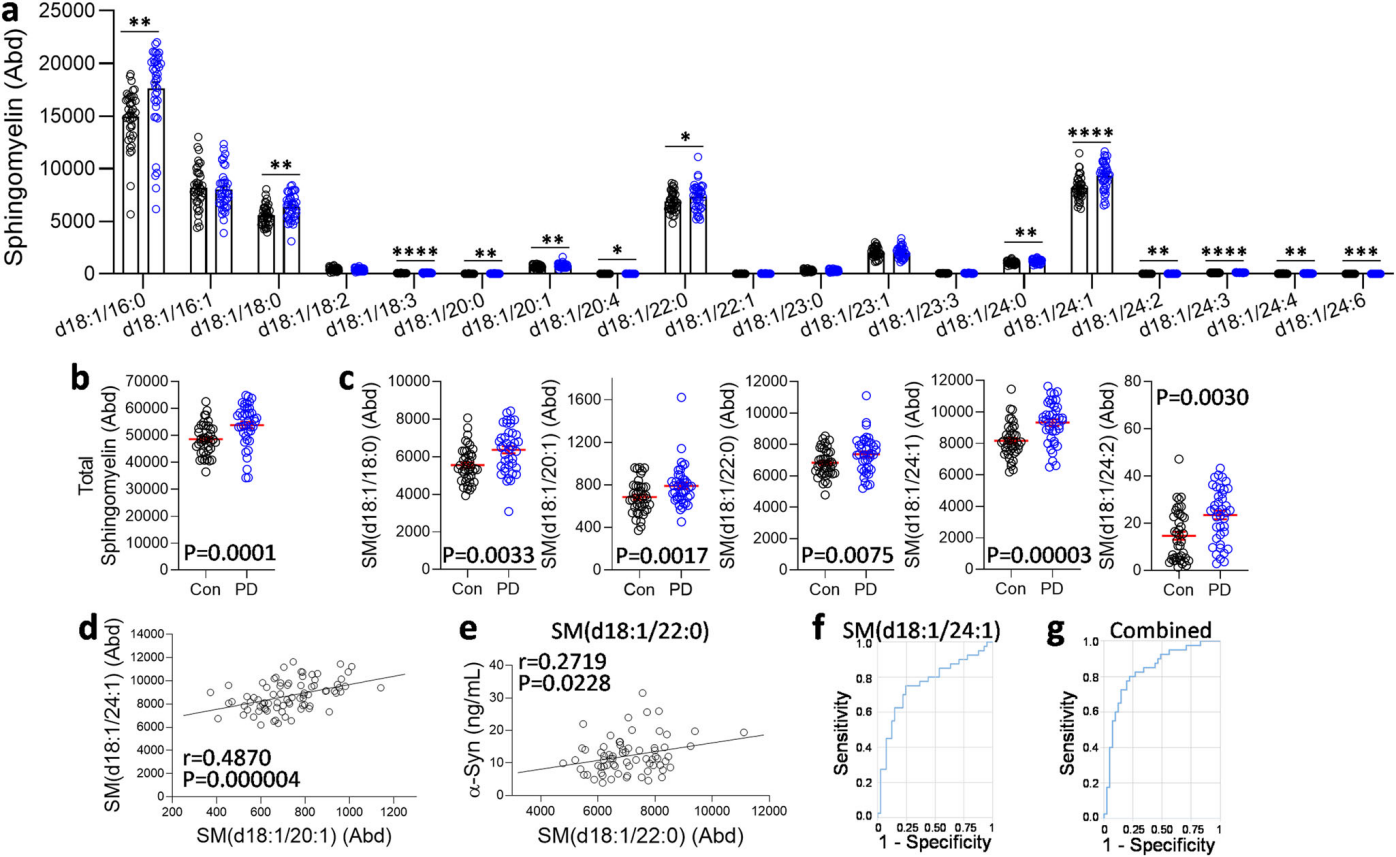
Liquid chromatography-mass spectrometry analysis of sphingomyelin in sporadic PD plasma. **a** Thirteen sphingomyelin species were increased in the PD plasma compared to controls. **b** The total sphingomyelin levels were also increased in the PD plasma. **c** Five identical sphingomyelin species were increased in both the PD plasma and the amygdala. **d** A representative example of the strong correlation among the five sphingomyelin species. **e** Sphingomyelin d18:1/22:0 levels correlated significantly with plasma α-synuclein levels. **f** Sphingomyelin d18:1/24:1 yielded the strongest ROC curve. **g** A combined ROC curve of the five species. Data represent mean and S.E.M. as error bars. *P < 0.05, **P < 0.01, ***P < 0.005, ****P < 0.001.

Since sphingomyelin levels were increased in PD brain, we were interested in whether sphingomyelin levels are also altered in the periphery and measured sphingomyelin in sporadic PD and control plasma using the same LC-MS method. Nineteen sphingomyelin species were detected and 13 were increased in PD compared to controls (Fig. 11a); total sphingomyelin levels were also increased in PD (Fig. 11b). Importantly, 5 sphingomyelin species – d18:1/18:0, d18:1/20:1, d18:1/22:0, d18:1/24:1 and d18:1/24:2 – were shown to be increased in both the plasma and the brain (Fig. 11c). These species were strongly correlated to each other (Fig. 11d). Interestingly, sphingomyelin d18:1/22:0 correlated with plasma α-synuclein levels (Fig. 11e). Finally, ROC curve analysis showed that sphingomyelin d18:1/24:1 was the strongest discriminator between PD and controls (AUC = 0.760; 75% sensitivity and 76% specificity) (Fig. 11f). A combined analysis of the five ROC curves yielded an improved AUC value of 0.819 (88% sensitivity and 71% specificity) (Fig. 11g).

## Discussion

Much evidence points to the dysregulation of sphingolipids in lysosomes in the pathogenesis of PD. Understanding the role of each of the LSP sphingolipid classes in the pathological processes of PD is limited or unknown. Clues to the pathological role of sphingomyelin in PD comes from a 1975 study that showed that sphingomyelin levels are increased in the brain of PD patients without levodopa treatment^20^. In the current study, we undertook a comprehensive assessment of the six LSP sphingolipid classes in the disease-affected amygdala and disease-unaffected visual cortex of sporadic PD brain. We found that ten sphingomyelin species and the total sphingomyelin levels were significantly increased in the PD amygdala compared to controls. Sphingomyelin levels were also correlated with disease duration only in the amygdala. No significant changes were detected in the visual cortex. The total levels of the five other sphingolipid classes were unchanged, although a few changes were detected at the species level. It appears that sphingomyelin is the mostly affected sphingolipid class in sporadic PD amygdala.

We chose amygdala as the disease-affected region of interest, because although this region undergoes severe pathological changes during the course of PD the neuronal integrity is mostly intact^19^ as confirmed by our NfL measurements. The accessory cortical and central nuclei of the amygdala are affected most, whereas the much larger basal and lateral nuclei and the intercalated cell masses are generally spared^21^. We intentionally avoided using the substantia nigra, because of severe neuronal cell loss to this region in PD.

Since polymorphisms in the ABCA5 gene appear protective against PD^15^ and the fact that ABCA5 is a lysosomal transporter that mediates the removal of excess sphingomyelin from lysosomes, we were interested in understanding if ABCA5 was changed in PD and measured the expression of ABCA5 in the same tissue samples as the lipid analysis. We found that ABCA5 expression was upregulated in the PD amygdala compared to controls, and it correlated strongly with sphingomyelin levels, which were also elevated in blood samples from PD patients. Furthermore, the in vitro studies using a neuronal model verified that ABCA5 expression is dependent on cellular levels of sphingomyelin. When put together, these results suggest a possible protective role of increased ABCA5 in response to elevated lysosomal sphingomyelin levels in PD that is then possibly eliminated from the brain via blood.

In terms of α-synuclein pathology, there was a strong association between sphingomyelin level and α-synuclein load in the disease-affected amygdala and no such association in the visual cortex. Consistent with our finding, a recent study reported that glucosylsphingosine, a sphingolipid, was strongly associated with a-synuclein pathology in disease-affected regions of PD brain^5^. Our cellular data showed that cellular sphingomyelin levels had a direct effect on α-synuclein expression, and it is known that increased α-synuclein levels leads to its aggregation^22^. It is also known that α-synuclein has the propensity to bind to lipid structures or lipid membranes that are enriched in sphingomyelin, and that the processing/turnover of α-synuclein is dependent on the sphingomyelin composition of lipid rafts^23–25^, so increased cellular sphingomyelin could trap α-synuclein. When put together, these results suggest that the transport of sphingomyelin from dysfunctional lysosomes is related to α-synuclein pathology, potentially via modulation of α-synuclein expression and/or sphingomyelin cellular concentrations.

Since ABCA5 could be playing a protective role by lowering elevated lysosomal levels of sphingomyelin in PD brain, we were interested in how ABCA5 is transcriptionally controlled. Members of ABCA transporter subfamily are typically regulated by nuclear receptor family of transcription factors. Transcriptional control of ABCA transporters has been shown to be important in a number of neurodegenerative processes. For example, promotion of ABCA1 transcription by LXR/RXR ligands stimulated cholesterol efflux^26^, and subsequently increased clearance of amyloid-β peptides^9^ and decreased amyloid-β deposition in the brain of AD mouse models^12,27^. In our study, ABCA5 transcription was clearly shown to be regulated by PPARγ/RXR, in a time- and dose-dependent manner. This is an important finding as this would provide a means for controlling ABCA5 function and therefore provide a possible pharmacological intervention against the elevated sphingomyelin levels observed in PD.

Of interest sphingomyelin levels, both individual species and total levels, were increased in sporadic PD plasma compared to controls. Importantly, five of these species were identical to those that were increased in the PD amygdala, suggesting they may be related. Based on the ROC curve analyses, these displayed some potential to discriminate PD from controls and could also be used to measure target engagement under pharmacologically treatments. Furthermore, sphingomyelin d18:1/22:0 was shown to be correlated with the plasma α-synuclein levels. These are important findings as they will provide a new avenue for the development of peripheral biomarkers to detect pathophysiological changes of relevance in PD.

A limitation of our study could be that the lipid analysis was carried out only in the amygdala and visual cortex. Therefore, future studies could include other regions of the brain that are affected by PD. Also of potential significance, further investigation is necessary to verify the association between ABCA5 polymorphisms and PD in a variety of populations and meta-analysis data. The pathological mechanisms underlying this association remains to be determined.

The discovery of ABCA5 as a possible protective gene for PD holds promise in understanding the sphingomyelin dysregulation observed in PD. In conclusion, ABCA5 appears to play a protective role in the removal of excess lysosomal sphingomyelin associated with increasing α-synuclein in affected PD brain regions. The increase in neuronal sphingomyelin itself might be a precipitating factor for α-synuclein aggregation, underscoring the importance of sphingomyelin pathway as a disease-modifying target. Our study provides new pathways and targets associated with α-synuclein aggregation in PD brain for possible therapeutic intervention and biomarker development for PD.

## Methods

### Human brain tissues

Frozen post-mortem brain tissue samples were obtained from Sydney Brain Bank and NSW Brain Tissue Resource Centre. Ethical approvals were acquired from the human research ethics committees of University of New South Wales (approval number: HC16568) and the University of Sydney (approval number: 2020/707). Frozen samples from the amygdala and the visual cortex from 8 PD and 10 controls without neurological, psychiatric or neuropathological diagnoses were used in this study. The mean age of PD and control groups were 80.3 and 78.9 years (*P* = 0.7087), respectively. The mean postmortem interval for the two groups were 20.4 and 23.9 hours (*P* = 0.4797), respectively.

### Human blood plasma

Participants were recruited with written informed consent and the study was approved by both the University of Sydney, and University of New South Wales, Research Ethics Committees (Approval numbers 2016/363 and HC16562, respectively). Participants with PD met the Movement Disorders Society criteria for clinically established PD^28^, and the control group comprised unaffected spouses of PD subjects and had no diagnosed neurological disease. This cohort has been described previously and used to study glucocerebrosidase activity in peripheral mononuclear cells^4^. Blood was collected from participants using BD CPT tubes following the manufacturers’ protocol, and plasma was snap-frozen in aliquots and stored at −80 °C until analysis.

### Chemicals and materials

Lipids were extracted using chloroform or methyl-t-butyl ether, methanol and isopropanol (Sigma Aldrich, St. Louis, MO, USA) and ultrapure water (Millipore). All solvents used were HPLC grade or higher. Glass pipettes and tubes were used wherever possible and the use of plasticware was minimized during lipid extraction to avoid contamination of samples. Glass tubes and glass transfer pipettes were purchased from Sigma and VWR. Lipid internal standards (ISTDs) were purchased from Avanti Polar Lipids Inc. (Alabaster, AL, USA). These include phosphatidylcholine (19:0), sphingomyelin (12:0), phosphatidylethanolamine (17:0), phosphatidylglycerol (17:0), phosphatidylserine (17:0), phosphatidic acid (17:0), ceramide (d18:1, 12:0), diglyceride (1,3 18:0 d5), cholesteryl ester (19:0), monoglyceride (17:0), triglyceride mix d5 (Avanti Code LM-6000), diglyceride mix d5 (Avanti Code LM-6001), phosphatidylinositol (17:0 14:1), C12 GluCer, C12 sulfatide, C17 ceramide, C17 sphingosine, C17 S1P, C12 C1P, D3 C20 fatty acid, and C12 LacCer. Lipid internal standards were prepared as a mixture at typically 10 pmol/µl in methyl-tert butyl ether and methanol (MTBE:methanol, 1:1 v/v).

### Immunohistochemistry and immunofluorescence

Formalin-fixed paraffin-embedded sections were deparaffinized with xylene and then rehydrated with gradient ethanol and water. Antigen retrieval was conducted with 70% formic acid followed by heating in a pressure cooker (Aptum Bio Retriever 2100, Aptum Biologics Ltd, UK). For immunohistochemistry, endogenous peroxidase activity was quenched with 1% H_2_O_2_ in 50% ethanol for 30 min and then washed in distilled water. Sections were further blocked with 5% normal horse serum prior to incubation in medium containing primary antibody. For single staining with immunohistochemistry, pan α-synuclein antibody (BD Transduction Laboratories™, Clone 42; 1:500) was applied. Sections were washed and incubated with the secondary antibody Horseradish Peroxidase-anti-Mouse, VECTOR ImmPRESS cat#MP-7402. The immunoreactive color was developed with ImmPACT DAB EqV Peroxidase (HRP) Substrate (VECTOR, SK-4103), prior to counterstaining with hematoxylin. Sections were then dehydrated with gradient ethanol and cleared with xylene prior to coverslipping with D.P.X. Images were obtained at x20 magnification using an Olympus slide scanner (VS120).

For immunofluorescence, after antigen retrieval, autofluorescence was quenched with LED light, followed by Sudan black B treatment. Sections were then incubated with the cocktail of primary antibodies against phospho-S129 (BioLegend 825701; 1:250), ABCA5 (Abcam ab99953; 1:100), and NeuN (antibodies.com, A270544; 1:100) at 4 °C for 2 days. After washing the sections were incubated with the corresponding secondary antibodies (donkey anti-goat Alexa Fluor 647, donkey anti-rabbit Alexa Fluor 568, donkey anti-mouse Alexa Fluor 488; 1:250) with 4′,6-diamidino-2-phenylindole (DAPI, Sigma cat# D9542, 1 mg/ml) at room temperature for 2 h. Sections were washed before being mounted with fluorescence mounting medium (DAKO, cat# S3023). Negative controls were performed by omitting either the primary or secondary antibodies. Images were obtained at x20 magnification using an Olympus slide scanner (VS120).

### Lipid extraction

Brain tissue lipid extraction was based on the Matyash method^29^. Briefly, 10 mg of fresh-frozen brain tissues were homogenized in methanol containing 0.01% BHT (300 µl) using a Qiagen TissueLyser (3 × 30 sec, 30 Hz cycles). The homogenates were transferred to glass tubes, as well as the methanol (430 µl) wash of the beads. MTBE (2.42 ml) was added and the mixture vortexed and incubated for 30 min at room temperature. Water (600 µl) was added and the mixture vortexed and centrifuged at 1000 *g* for 10 min. The upper phase was transferred to a new glass tube using a glass Pasteur pipette. The lower phase was re-extracted using MTBE/MeOH/water (10:3:2.5). The combined extracts were dried under nitrogen gas. Dried lipid samples were reconstituted in 100 µl of chloroform/methanol (1:1) and stored at −80 °C in glass LC-MS vials.

Plasma lipid extraction was based on the Bligh and Dyer method^30^. Briefly, plasma samples were thawed on ice and 80 µl aliquots were transferred into glass tubes. Methanol (600 µl), chloroform (1,000 µl) and ultrapure water (500 µl) were sequentially added with vortexing between each addition. Samples were then centrifuged at 3,000 rpm for 10 min at room temperature. The lower solvent phase was collected and transferred to a new glass tube using a glass Pasteur pipette. Chloroform (600 µl) was added, vortexed and centrifuged at 3,000 rpm for 10 min. The lower phase was collected, combined with the previous extract and dried under nitrogen gas. Dried lipid samples were reconstituted in 100 µl of isopropanol/methanol (1:1) and stored at −80 °C in glass LC-MS vials.

### Liquid chromatography-mass spectrometry

Lipid extracts (10 μl) were analyzed using a Q-Exactive Plus Mass Spectrometer coupled to a U3000 UPLC system (ThermoFisher Scientific). Chromatography was performed at 60° C on a Waters CSH C18 UHPLC column 2.1 × 100 mm, 1.8 μM with VanGuard guard column. Solvent A was 6:4 acetonitrile:water and Solvent B was 1:9 acetonitrile:isopropanol, both with 10 mM ammonium formate and 0.1% formic acid. Lipids were chromatographed according to the method of Castro-Perez et al.^31^. Briefly, a 30 min gradient running from 30 to 100% of solvent B was performed, eluting lipids in order of hydrophobicity. Column eluate was directed into the electrospray ionization source of the mass spectrometer where a HESI probe was employed. Source parameters were broadly optimized on a range of lipid standards prior to the analysis. The mass spectrometer was run in data-dependent acquisition mode. A survey scan over the mass range 200–1,200 at resolution 70 K was followed by 10 data-dependent MS/MS scans on the most intense ions in the survey at 15 K resolution. Dynamic exclusion was used to improve the number of ions targeted. Cycle time was approximately 1 sec. Samples were run in both positive and negative polarities. The samples were run in a random order (generated using Microsoft Excel). This is important to avoid batch effects/changing instrument performance effects. Data were analysed in LipidSearch software 4.1.16. Data were searched against the standard Lipidsearch database with all common mammalian lipid classes included. The search results were then grouped according to sample type and aligned for differential analysis. Aligned data (containing lipid identity, peak area, retention time, etc.) were exported to Excel software. Abundance of lipids was obtained from peak areas for each lipid species. They were normalized between samples, to correct for batch effects from the sample preparation and the LC-MS/MS analysis, using the internal standards of the same lipid category. They were then normalized to the weight of the brain tissues used.

### Protein extraction

Protein was serially extracted from 100 mg of fresh-frozen brain tissues as previously described^32^. Briefly, tissues were homogenized in ten volumes of TBS homogenization buffer (20 mM Tris, 150 mM NaCl, pH 7.4, 5 mM EDTA, 0.02% sodium azide) containing protease inhibitor cocktail (Roche) using Qiagen TissueLyser (3 × 30 sec, 30 Hz cycles), followed by centrifugation at 100,000 *g* for 1 h at 4 °C. The pellet was resuspended in TBS homogenization buffer containing 5% SDS using TissueLyser (30 Hz cycles, 3×30 sec), centrifuged at 100,000 *g* for 30 min at 25 °C and supernatant collected. Protein concentration on the supernatant was measured using a bicinchoninic acid assay (Pierce BCA Protein Assay Kit) following the manufacturer’s instructions.

### Western blotting and ELISA

Protein lysates (15 µg) were heated with sample buffer (3.2% SDS, 32% glycerol, 0.16% bromophenol blue, 100 mM Tris-HCl, pH 6.8, 8% 2-mercaptoethanol). They were then electrophoresed on Criterion Stain-free 4-20% SDS-PAGE gels (Bio-Rad) and transferred onto nitrocellulose membranes at 100 volts for 30 min. The membranes were blocked with TBS containing 5% nonfat dry milk and probed with α-synuclein antibody (1:1000, BD Biosciences, 610787) or NfL antibody (1:2000, Cell Signaling, 2835 S) overnight at 4 °C. They were then washed three times in TBS containing 0.1% Tween 20 and incubated with horseradish peroxidase-conjugated secondary antibody for 2 h at room temperature. Signals were detected using enhanced chemiluminescence and Gel Doc System (Bio-Rad). The blots were stripped and probed for housekeeper proteins GAPDH or β-actin. The signal intensity was quantified using Image Lab (Bio-Rad) and NIH ImageJ software (v1.45 s). All blots were derived from the same experiment and that they were processed in parallel. ELISA of plasma α-synuclein (Legend Max #844101) was carried out following the manufacturer’s protocol.

### Cell studies

SK-N-SH neuronal cells were obtained from the ATCC (Manassas, VA). Primary human fetal brain cells were a kind gift from Prof Guillemin and were prepared as described previously^33–35^, and the study was approved by the University of New South Wales Human Research Ethics Committee (approval number: HREC 03187). Cells were cultured in Dulbecco’s modified Eagle’s medium containing 10% fetal calf serum, 1% Glutamax, 0.5% glucose, 100 IU/ml penicillin and 100 μg/ml streptomycin at 37 °C in humidified air containing 5% CO_2_. SK-N-SH cells were transfected with ABCA5 cDNA or empty vector (control) using Lipofectamine 2000 (Thermo Fisher Scientific) following the manufacturer’s protocol. After 48 hours the cells were harvested and total RNA prepared for gene expression studies. In the sphingomyelin study, cells were cultured in 12-well plates and treated with 40 µM sphingomyelin (Sigma-Aldrich #85615), 50 µM D609 inhibitor or vehicle (control) for 24 hours. In the regulation study, cells were cultured in 12-well plates and treated with 10 µM T0901317, 10 µM 9-cis retinoic acid, 10 µM pioglitazone (Sigma-Aldrich) or vehicle (control) for 24 h. The cells were harvested and total RNA prepared for gene expression analysis.

### RNA extraction and quantitative PCR

RNA was isolated using TRIzol reagent (Invitrogen) following the manufacturer’s protocol as previously described^36^. All procedures were carried out using RNase-free reagents and consumables. One microgram of RNA was reverse transcribed into cDNA using Moloney-murine leukemia virus (M-MLV) reverse transcriptase and random primers (Promega, Madison, Wisconsin, USA) in 20 μl reaction volume. Quantitative PCR (qPCR) assays were carried out using a Mastercycler ep realplex S (Eppendorf, Sydney, Australia) and the fluorescent dye SYBR Green (Bio-Rad), following the manufacturer’s protocol. Briefly, each reaction (20 μl) contained 1x mastermix, 5 pmoles of primers and 1 μl of cDNA template. Amplification was carried out with 40 cycles of 94 °C for 15 s and 60 °C for 1 min. Gene expression was normalized to the geometric mean of three housekeeper genes, GAPDH, β-actin and PPIA. A no-template control was included for each PCR amplification assay. The level of expression for each gene was calculated using the comparative threshold cycle (Ct) value method using the formula 2^−ΔΔCt^ (where ΔΔCt = ΔCt sample – ΔCt reference).

### Statistical analysis

Statistical analyses were performed using SPSS Statistics software (IBM, Chicago, Illinois) as previously described^36^. Multivariate analyses (general linear model) covarying for age and sex were used to determine differences in lipid levels in PD and control groups with posthoc statistical significance set at *p* < 0.05. Pearson’s correlations were used to determine if changes in measurements were associated with each other with statistical significance set at *p* < 0.05. Receiver operating characteristic (ROC) curve was generated for each sphingomyelin species and CombiROC^37^ for a combination of the five sphingomyelin species to discriminate PD from controls. Graphs were generated using GraphPad Prism 9.

### Supplementary information

nr-reporting-summary

## Data Availability

Lipidomics raw data were generated at Bioanalytical Mass Spectrometry Facility, University of New South Wales. Derived data supporting the findings of this study are available from the corresponding author. Other patient data cannot be made publicly available because the ethical approval and the written informed consent from the patients included in this study did not cover placing the data into publicly open repositories.
