# Supplementary material for: A protective role of ABCA5 in response to elevated sphingomyelin levels in Parkinson’s disease
Source: NPJ Parkinsons Dis. 2024 Jan 11;10:20. doi: 10.1038/s41531-024-00632-2 (PMC10784510; doi:10.1038/s41531-024-00632-2)
Supplement: Supplementary file 1 — nr-reporting-summary [file 41531_2024_632_MOESM1_ESM.pdf]

nature portfolio

Corresponding author(s): Woojin Kim

Last updated by author(s): Nov 22, 2023

Reporting Summary

Nature Portfolio wishes to improve the reproducibility of the work that we publish. This form provides structure for consistency and transparency in reporting. For further information on Nature Portfolio policies, see our Editorial Policies and the Editorial Policy Checklist.

Statistics

For all statistical analyses, confirm that the following items are present in the figure legend, table legend, main text, or Methods section.

n/a	Confirmed	
☐	☒	The exact sample size (*n*) for each experimental group/condition, given as a discrete number and unit of measurement
☐	☒	A statement on whether measurements were taken from distinct samples or whether the same sample was measured repeatedly
☐	☒	The statistical test(s) used AND whether they are one- or two-sided<br>*Only common tests should be described solely by name; describe more complex techniques in the Methods section.*
☐	☒	A description of all covariates tested
☒	☐	A description of any assumptions or corrections, such as tests of normality and adjustment for multiple comparisons
☒	☐	A full description of the statistical parameters including central tendency (e.g. means) or other basic estimates (e.g. regression coefficient) AND variation (e.g. standard deviation) or associated estimates of uncertainty (e.g. confidence intervals)
☐	☒	For null hypothesis testing, the test statistic (e.g. *F*, *t*, *r*) with confidence intervals, effect sizes, degrees of freedom and *P* value noted<br>*Give P values as exact values whenever suitable.*
☒	☐	For Bayesian analysis, information on the choice of priors and Markov chain Monte Carlo settings
☒	☐	For hierarchical and complex designs, identification of the appropriate level for tests and full reporting of outcomes
☐	☒	Estimates of effect sizes (e.g. Cohen's *d*, Pearson's *r*), indicating how they were calculated

Our web collection on statistics for biologists contains articles on many of the points above.

Software and code

Policy information about availability of computer code

Data collection	Nikon NIS Elements software (version 4.60)
Data analysis	SPSS Statistics software 26 (IBM, Chicago, Illinois)

For manuscripts utilizing custom algorithms or software that are central to the research but not yet described in published literature, software must be made available to editors and reviewers. We strongly encourage code deposition in a community repository (e.g. GitHub). See the Nature Portfolio guidelines for submitting code & software for further information.

Data

Policy information about availability of data

All manuscripts must include a data availability statement. This statement should provide the following information, where applicable:

- Accession codes, unique identifiers, or web links for publicly available datasets
- A description of any restrictions on data availability
- For clinical datasets or third party data, please ensure that the statement adheres to our policy

All data analyzed are included in this manuscript and are available from the corresponding author upon request.

Research involving human participants, their data, or biological material

Policy information about studies with human participants or human data. See also policy information about sex, gender (identity/presentation), and sexual orientation and race, ethnicity and racism.

Reporting on sex and gender	Sex was used as a co-variate in the statistical analysis only.
Reporting on race, ethnicity, or other socially relevant groupings	Not applicable
Population characteristics	Not applicable
Recruitment	Not applicable
Ethics oversight	University of New South Wales (approval number: HC16568) and the University of Sydney (approval number: 2020/707)

Note that full information on the approval of the study protocol must also be provided in the manuscript.

Field-specific reporting

Please select the one below that is the best fit for your research. If you are not sure, read the appropriate sections before making your selection.

☒ Life sciences ☐ Behavioural & social sciences ☐ Ecological, evolutionary & environmental sciences

For a reference copy of the document with all sections, see nature.com/documents/nr-reporting-summary-flat.pdf

Life sciences study design

All studies must disclose on these points even when the disclosure is negative.

Sample size	8 Parkinson's disease and 10 control tissue samples were used in this study. No sample size calculations were performed. Previous data using the same methods to measure lipids in samples from dementia cases (N=5 or 8 per group) found an average 25-30% increase in lipid levels (J Lipid Res 45:186-193).
Data exclusions	No data were excluded from the analyses.
Replication	All assays (i.e. lipid, western blotting, qPCR etc) were performed on all cases, i.e. 8 Parkinson's disease and 10 control) and all data presented.
Randomization	In the mass spectrometry, the samples were run in a random order to avoid batch effects/changing instrument performance effects.
Blinding	The investigators were blinded in the mass spectrometry data acquisition.

Reporting for specific materials, systems and methods

We require information from authors about some types of materials, experimental systems and methods used in many studies. Here, indicate whether each material, system or method listed is relevant to your study. If you are not sure if a list item applies to your research, read the appropriate section before selecting a response.

Materials & experimental systems

n/a	Involved in the study	
☐	☒	Antibodies
☐	☒	Eukaryotic cell lines
☒	☐	Palaeontology and archaeology
☒	☐	Animals and other organisms
☒	☐	Clinical data
☒	☐	Dual use research of concern
☒	☐	Plants

Methods

n/a	Involved in the study	
☒	☐	ChIP-seq
☒	☐	Flow cytometry
☒	☐	MRI-based neuroimaging

Antibodies

Antibodies used	pan α-synuclein antibody (BD Transduction Laboratories, Clone 42; 1:500); phospho-S129 (BioLegend 825701; 1:250); ABCA5 (Abcam ab99953; 1:100); NeuN (antibodies.com, A270544; 1:100); phospho-S129 (BioLegend 825701; 1:250); ABCA5 (Abcam ab99953;

1:100); and NeuN (antibodies.com, A270544; 1:100); α-synuclein antibody (1:1000, BD Biosciences, 610787); NfL antibody (1:2000, Cell Signaling, 2835S)

Validation	Validation information are available on manufacturer's website.

Eukaryotic cell lines

Policy information about cell lines and Sex and Gender in Research

Cell line source(s)	SK-N-SH cell line was obtained from ATCC (Manassas, VA).
Authentication	SK-N-SH cell line was not authenticated.
Mycoplasma contamination	SK-N-SH cell line was tested negative for mycoplasma contamination.
Commonly misidentified lines (See ICLAC register)	No commonly misidentified lines were used.

Plants

Seed stocks	N/A
Novel plant genotypes	N/A
Authentication	N/A
